# Characteristics of COVID-19 Disease in Renal Transplant Recipients

**DOI:** 10.3390/medicina60020201

**Published:** 2024-01-24

**Authors:** Emilija Zimnickaitė, Ieva Kucinaitė, Birutė Zablockienė, Aistė Lisinskaitė, Rolandas Zablockis, Laurynas Rimševičius, Marius Miglinas, Ligita Jančorienė

**Affiliations:** 1 Faculty of Medicine, Vilnius University, M. K. Ciurlionio 21, 03101 Vilnius, Lithuania; 2 Clinic of Infectious Diseases and Dermatovenerology, Institute of Clinical Medicine, Faculty of Medicine, Vilnius University, M. K. Ciurlionio 21, 03101 Vilnius, Lithuania; birute.zablockiene@santa.lt; 3 Center of Infectious Diseases, Vilnius University Hospital Santaros Klinikos, Santariskiu Street 14, 08406 Vilnius, Lithuania; 4 Clinic of Chest Diseases, Immunology and Allergology, Institute of Clinical Medicine, Faculty of Medicine, Vilnius University, M. K. Ciurlionio 21, 03101 Vilnius, Lithuania; rolandas.zablockis@santa.lt; 5 Clinic of Gastroenterology, Nephrourology and Surgery, Institute of Clinical Medicine, Faculty of Medicine, Vilnius University, M. K. Ciurlionio 21, 03101 Vilnius, Lithuania

**Keywords:** SARS-CoV-2, kidney transplantation, Charlson comorbidity index, mortality

## Abstract

*Background and Objectives:* Kidney transplant recipients are at risk of developing more severe forms of COVID-19 infection. The aim of this study was to compare the clinical course of COVID-19 infection among kidney transplant patients and a control group. *Materials and Methods:* We examined 150 patients hospitalized with COVID-19 infection. Patients were divided into study (kidney transplant recipients, *n* = 53) and control (without a history of kidney transplantation, *n* = 97) groups. Demographics, clinical characteristics, treatment data, and clinical outcomes were assessed. *Results:* The median patient age was 56.0 (46.0–64.0) years, and seventy-seven patients (51.3%) were men. The median Charlson comorbidity index was higher in the study group (3.0 vs. 2.0, *p* < 0.001). There was a higher incidence of hypoxemia in the control group upon arrival (52.6% vs. 22.6%, *p* = 0.001) and a higher NEWS index median (2.0 vs. 1.0 points, *p* = 0.009) and incidence of pneumonia during hospitalization (88.7% vs. 73.6%, *p* = 0.023). In the study group, there were more cases of mild (26.4% vs. 11.3%, *p* = 0.023) and critically severe forms of COVID-19 infection (26.4% vs. 3.1%, *p* < 0.001), kidney failure was more prevalent (34.0% vs. 1.0%, *p* < 0.001), and a greater number of patients were transferred to the intensive care unit (22.6% vs. 3.1%, *p* < 0.001) and died (18.9% vs. 1.0%, *p* < 0.001). Multivariable analysis revealed that treatment in the intensive care unit correlated with a higher mortality rate than transplantation itself (HR = 20.71, 95% CI 2.01–213.33, *p* = 0.011). *Conclusions:* The course of the COVID-19 disease in kidney transplant recipients is heterogeneous and can be more severe than in the general population. Even though patients may be hospitalized with fewer symptoms, complications and death are more likely to occur.

## 1. Introduction

Coronaviruses are a family of single-stranded RNA viruses named for their distinctive surface protein structure, which resembles the outer part of the solar atmosphere, the solar corona [1].

On 12 December 2019, the first patient with pneumonia of unknown etiology was hospitalized in Wuhan, China [2], and on 11 March 2020, the WHO declared COVID-19 a pandemic [3]. To date, 763 million people have contracted COVID-19, of whom 6.9 million have died [4].

COVID-19 infection is characterized by upper and lower respiratory tract infection symptoms: cough, fever, muscle and/or headache, shortness of breath, and loss of taste/smell [5]. Pathophysiologically, there is a fierce ‘battle’ between the infectious SARS-CoV-2 virus and the human immune system. It has been reported that patients with COVID-19 infection have systemic hyperinflammatory reactions characterized by the release of proinflammatory cytokines (interleukins (IL)-1, IL-6, IL-8, and tumor necrosis factor (TNF)) and increased serum concentrations of proinflammatory markers (D-dimers, ferritin, and C-reactive protein (CRP)). The severe form of COVID-19 can also lead to extrapulmonary manifestations of the disease, including gastrointestinal symptoms, acute cardiac, renal, or hepatic damage, as well as cardiac rhythm disturbances, rhabdomyolysis, coagulopathies, and shock [6,7].

Individuals with immune deficiencies are particularly susceptible to COVID-19 infection [8]. A typical example is patients who have undergone solid organ transplants (SOT), the most common of which is kidney transplantation [9]. Any exposure to infectious agents can be particularly dangerous due to the immunosuppressive therapy administered to reduce the risk of graft rejection while, at the same time, decreasing the effectiveness of the immune response [10]. The leading causes of death among them are infectious diseases and their complications [11].

The aim of this study was to compare the clinical course of COVID-19 infection in kidney transplant patients and controls. We evaluated the differences in demographic characteristics and risk factors for the severe disease, clinical data, laboratory and instrumental tests during the course of the hospitalization, treatment administered, complications, and outcomes between research and control groups. 

## 2. Materials and Methods

Subjects: The retrospective study included data from 150 patients hospitalized for COVID-19 infection at the Infectious Disease ward of Vilnius University Hospital Santaros Clinics, Lithuania, between July 2020 and April 2022. Patients were divided into control (*n* = 97) and study (*n* = 53) groups. The latter included patients with a history of renal transplantation. The vaccination and immune response status were investigated only in the control group. All immunizations were performed using the “Comirnaty” mRNA vaccine. The development of humoral immunity was assessed by the production of IgG class antibodies against SARS-CoV-2 in blood serum.

Methods

Demographic characteristics: The Charlson comorbidity score (CCS), a predictive index assessing the 10 year survival probability of a patient with comorbid chronic diseases, was used to determine the degree of comorbidity [12].

Assessment of disease severity: Standardized condition monitoring is provided by the National Early Warning Score (NEWS), a composite indicator calculated by assessing the patient’s respiratory rate, oxygen saturation, systolic blood pressure, pulse, level of consciousness, and body temperature [13]. Hypoxemia is defined when oxygen saturation (SpO_2_) measured by pulse oximetry is <94%. The diagnosis of acute kidney injury is based on the Kidney Disease: Improving Global Outcomes (KDIGO) guidelines [14]. The severity of COVID-19 is determined by the order of the Minister of Health of the Republic of Lithuania (hereinafter referred to as “the Minister of Health”) “On the approval of the description of the procedure for diagnosis and treatment of COVID-19 disease (Coronavirus infection) in children and adults” [15], which is based on National Institutes of Health guidelines [16]. Mild: uncomplicated upper respiratory tract infection; moderate: pneumonia without signs of severe pneumonia; severe: pneumonia with signs of hypoxemia requiring oxygen treatment; critically severe: sepsis, septic shock, acute respiratory distress syndrome (ARDS), or multiple organ dysfunction syndrome (MODS). Indications for intensive care unit (ICU) treatment include failure to achieve target oxygenation levels while administering respiratory corrective therapy, the patient requiring treatment with invasive mechanical ventilation (IMV), the patient developing shock, or the patient developing a critical condition due to organ system dysfunction [15]. Patients treated in the ICU received high-flow oxygen therapy (HFOT), non-invasive ventilation (NIV), and/or invasive mechanical ventilation (IMV). 

Laboratory data: All patients underwent comprehensive laboratory tests during admission and hospitalization, e.g., automated blood and biochemical blood tests. In the present study, we analyzed leukocyte, neutrophil, and lymphocyte counts (×10^9^/L), inflammatory markers (CRB (mg/L), IL-6 (ng/L)) and renal function indices (creatinine (μmol/L), glomerular filtration rate (calculated) (Engl. estimated glomerular filtration rate (eGFR) (ml/min/1.73 m^2^), urea (mmol/L)) and ferritin (μg/L) concentrations, lactate dehydrogenase (LDH) activity (U/L), and D-dimer concentration (μg/L).

Methodology: The study evaluated demographic, clinical, and laboratory variables, treatment strategies, and outcomes. Data were collected from the electronic medical record (EMR) and the Electronic Health Services and Collaborative Infrastructure Information System (ESPBI IS). SARS-CoV-2 infection was detected by real-time polymerase chain reaction (PCR). The diagnosis of pneumonia was confirmed by radiological methods such as lateral and posteroanterior/anteroposterior chest radiographs or chest computed tomography (CT). The study was approved by the Vilnius Regional Biomedical Research Ethics Committee No. 2020/6-1233-718. 

Statistical analysis: The data were structured using Microsoft Excel 2013 and analyzed using IBM SPSS Statistics version 21.0. The quantitative variables are presented as mean and standard deviation (SD) (normal distribution) or median and interquartile range (IQR) (non-normal distribution), according to the Kolmogorov–Smirnov test. Frequency tables (cross tabs) were used to describe the qualitative variables, and the chi-square test and Fisher’s exact test were used for comparison. Variables with a *p*-value < 0.2 in the univariate regression analysis were included in the multivariate analysis with a hazard ratio (HR) and confidence interval (CI). In all analyses, the difference between variables was considered statistically significant at *p* < 0.05. 

## 3. Results

In total, data from 150 patients were analyzed. The median age was 56.0 years, with a slightly lower median age in the study group (54.0 vs. 57.0 years, *p* = 0.043). Seventy-seven subjects (51.3%) were male. The median Charlson comorbidity index median was 2.0 in all subjects, with a significant between-group difference, with a higher score median in the study group (3.0 vs. 2.0, *p* < 0.001). Moreover, when comorbidities were analyzed separately, they were more frequent in the study group: arterial hypertension (96.2% vs. 57.7%, *p* < 0.001), chronic heart failure (41.5% vs. 16.5%, *p* = 0.001), gout (43.4% vs. 8.2%, *p* < 0.001), and diabetes mellitus (26.4% vs. 11.3%, *p* < 0.023) (Table 1).

The study group consisted of 53 individuals. The majority of them (90.4%) underwent one kidney transplantation and received cadaver kidney transplants (86.8%). The most common causes of renal failure requiring kidney transplantation were renal polycystosis (17.0%), primary glomerulonephritis (15.1%), and hypertensive nephropathy (13.2%). An unknown cause of kidney damage was found in fifteen cases (transplantation > 20 years ago): unspecified glomerulonephritis (17.0%) and unspecified chronic kidney disease (11.3%). The median time from the last transplant to hospitalization for COVID-19 infection was 5.0 years (Table 2).

The median time from symptom onset to hospitalization was 7.0 days (Table 3). Hypoxemia on admission was diagnosed in 63 (42.0%) patients, with a higher prevalence in the control group (52.6% vs. 22.6%, *p* < 0.001), as well as higher NEWS scores (2.0 vs. 1.0 points, *p* = 0.009). Pneumonia was more frequently diagnosed in the control group during hospitalization (88.7% vs. 73.6%, *p* = 0.023). The incidence of COVID-19 disease forms also differed between the control and study groups (*p* < 0.001): the incidence of a severe form of the disease was higher in the control group (70.1% vs. 32.1%, *p* < 0.001), but the incidence of both mild (26.4% vs. 11.3%, *p* = 0.023) and critically severe forms was higher in the study group (26.4% vs. 3.1%, *p* < 0.001). Nineteen patients (12.7%) developed acute kidney injury during hospitalization, with a higher prevalence in kidney transplant patients (34.0% vs. 1.0%, *p* < 0.001) (Table 3).

Patients in the study group tended to have higher laboratory parameters, showing kidney dysfunction during both admission and during the hospitalization. (detailed information can be found in Table 4).

The median length of hospitalization was 12.0 days. One hundred and five patients (70.0%) required oxygen therapy during hospitalization. Fifteen patients (10.0%) were treated in the intensive care unit. The study group was more likely to be treated in an intensive care unit (22.6% vs. 3.1%, *p* < 0.001). More detailed information on treatment can be found in Table 5. Eleven patients (7.3%) died, including a greater proportion of kidney transplant recipients (18.9% vs. 1%, *p* < 0.001) (Table 5). A multivariate regression analysis revealed that kidney transplantation was not associated with a lethal outcome in the case of COVID-19 infection (HR = 2.95, 95% CI 0.26–33.40), *p* = 0.383). Treatment in the intensive care unit was the only factor that correlated with a lethal outcome (HR = 20.71, 95% CI: 2.01–213.33), *p* = 0.011) (Table 6). 

Forty (75.5%) patients in the study group were vaccinated with the Comirnaty vaccine: one (1.9%) patient with one dose, thirteen (24.5%) with two doses, and twenty-six (49.1%) with three doses. Five patients (9.4%) received no vaccination; the immunization status of the rest (15.1%) is not known. Of the vaccinated patients, only nine patients (22.5%) developed antibodies against SARS-CoV-2: two (5.0%) and seven (17.5%) patients developed IgG antibodies after two and three doses of the vaccine, respectively. The immune response to vaccination of eight patients (20%) is not known.

## 4. Discussion

It is important to understand that renal transplant patients have a variety of comorbid conditions. Kidney transplant patients are often diagnosed with arterial hypertension, heart failure, coronary heart disease, diabetes mellitus, and gout [17]. As a result of aging and/or chronic kidney disease, these diseases are often developed before the transplantation itself; however, they may also develop as complications following the transplant [18] and are associated with higher mortality [19]. In our study, this tendency is also evident, with a higher Charlson comorbidity index in the study group compared to the control group, which is regarded as a valid prognostic indicator suggestive of a higher mortality rate in patients hospitalized for COVID-19 infection [20]. 

Even in the general population, comorbidities are associated with higher mortality [21]. A study in France found that patients with kidney transplants and comorbidities are also at increased risk of SARS-CoV-2 mortality [22]. In Canada, researchers observed that both immunosuppression and comorbidities are associated with a higher need for oxygen therapy, i.e., a more severe form of COVID-19 [23]. Some studies suggest that it is the co-existing comorbidities, rather than the intensity of immunosuppressive therapy administered, that is the more important prognostic factor for the course and mortality of COVID-19 [24,25]. In summary, there is currently still no scientific consensus on whether multimorbidity, immunosuppressive therapy, or a combination of both are factors contributing to the more severe course of COVID-19 infection. 

Immunosuppressive therapy in solid organ transplant patients often leads to delayed or atypical infectious symptoms, making timely diagnosis more difficult [10]. Data in the literature found that fever was less common in renal transplant recipients during COVID-19 infection [26]. Based on our analysis, the study group had lower NEWS values, fewer signs of respiratory failure, and was diagnosed with fewer cases of pneumonia at the time of admission compared to the control group; however, they developed more severe disease during hospitalization. The study group had a higher incidence of critically severe disease, a statistically significantly higher incidence of acute renal failure, and a higher incidence of ICU treatment, which is in line with the findings of publications by foreign colleagues [27,28,29,30,31]. The available information and comparison with the findings of studies by foreign authors suggest that SOT recipients have a rapidly progressive and unpredictable disease course [26]. As a result, medical experts in various countries have emphasized in publications and treatment guidelines that transplant recipients are of the greatest concern in terms of the COVID-19 pandemic [23,32].

Current data suggest that the overall mortality rate from COVID-19 infection is 0.7% in Lithuania [33] and 0.9% worldwide [4]. Based on the comparison of these two rates, we observed a significantly higher rate in the study group than the control group (18.9% vs. 1.0%, *p* < 0.001), as well as with the Lithuanian and global data. Numerous authors have described similar findings in their publications [28,34,35,36,37]. However, sporadic articles report that the mortality rate of COVID-19 infection in kidney transplant recipients is comparable to the general population [38]. Unfortunately, this aspect remains unsubstantiated in studies.

In our study, we found no correlation between kidney transplantation and lethal outcomes in COVID-19 disease. Only the treatment in the intensive care unit (i.e., a more severe course of the disease) significantly correlates with a higher mortality rate, but this is a known non-causal association. Similar results were reported in a multicenter study of liver transplant recipients with COVID-19 infection, where higher mortality was not due to liver transplantation per se, but to the older age of the patients and the severity of the disease [39].

The vaccination status in our study was analyzed only in the treatment group. Unfortunately, with the small sample size, even less data were found on the post-vaccination status of the patients, so no statistically reliable conclusions can be drawn. However, our results reflect a general trend that immune responses are relatively rare in vaccinated patients undergoing kidney transplantation. Clinical trials show that the efficacy of vaccines against COVID-19 infection is 91–94% in the general population [40]. However, COVID-19 vaccines are not as effective in solid organ transplant recipients receiving immunosuppressive therapy [41]. According to the literature, only approximately 37.5–52.4% of patients after kidney transplantation and vaccinated with two doses of the vaccine develop an immune response against COVID-19 infection [42,43]. 

Several potential risk factors for failed seroconversion have been suggested, immunosuppression and its intensity in the post-transplant period being the most important [44]. The number of vaccine doses received also influences the development of an immune response. If seroconversion does not occur after two doses, positive results are observed after the third [45] or even the fourth dose [46]. For this reason, booster doses are recommended for all solid organ transplant patients [23,47]. The clinical utility of seroconversion diagnostics is ambiguous. On the one hand, some authors recommend monitoring the humoral immune response and adapting vaccination protocols accordingly [48]. Nevertheless, large clinical reviews suggest that the benefit of repeated vaccination is questionable and that routine monitoring of the resulting immune response is not recommended [41,49]. Nevertheless, the benefit of vaccines, in general, is undeniable, with the resulting immune response leading to a lower incidence of treatment in the ICU and mortality in COVID-19 [50,51].

Our study has several limitations. Firstly, the retrospective nature of the study meant that we were faced with a lack of certain clinical indicators in the medical data systems and their interpretability. The most important of these are unknown/undocumented renal pathology leading to kidney transplantation in patients who underwent transplantation more than 15–20 years ago. Secondly, the study was conducted in only one center, which resulted in a limited sample of patients and, therefore, a narrow range of applicability of the results. Thirdly, it was not possible to assess the disease course of the patients according to the SARS-CoV-2 genomic variant, as this information was not identified in routine PCR testing. We would also like to point out that the severity of COVID-19 infection was classified according to the order of the Ministry of Health of the Republic of Lithuania into four forms (mild, moderate, severe, and critically severe) and differed from some foreign publications, which distinguished only three forms (mild, moderate, and severe). Moreover, patients started to be included in the study in July 2020 when there was no vaccine against COVID-19 infection, so a large part of the included subjects were not vaccinated at all. Some of the patients with transplanted kidneys received varying amounts of vaccine, all showing questionable immune responses. Furthermore, the use of CCI should be regarded with consideration, as the mere fact of kidney transplantation increases the value of this index. Therefore, we also evaluated the significance of individual diseases (arterial hypertension, chronic heart failure, gout, and diabetes mellitus) in the study groups. Finally, we could not reliably assess the intensity and effectiveness of the immunosuppressive treatment received by the study group on an outpatient basis and, therefore, its impact on the course of the disease.

## 5. Conclusions

The clinical course of COVID-19 infection in kidney transplant recipients and the control group was compared. The data reveal that kidney transplant recipients were often hospitalized with fewer symptoms, although they had a more severe course of the disease (higher rate of acute kidney injury and abnormal laboratory values). Additionally, the study group were more frequently treated in intensive care units, resulting in a higher mortality rate. 

## Figures and Tables

**Table 1:** Patient demographic characteristics.

	All Participants (*n* = 150)	Control Group (*n* = 97)	Study Group (*n* = 53)	*p*-Value
Age, years	56.0 (46.0–64.0)	57.0 (47.5–65.0)	54.0 (43.5–61.0)	**0.043**
Male sex	77 (51.3%)	50 (51.5%)	27 (50.9%)	0.540
CSS	2.0 (1.0–4.0)	2.0 (0.0–2.5)	3.0 (2.0–5.0)	**<0.001**
Arterial hypertension	107 (71.3%)	56 (57.7%)	51 (96.2%)	**<0.001**
Chronic heart failure	38 (25.3%)	16 (16.5%)	22 (41.5%)	**0.001**
Gout	31 (20.7%)	8 (8.2%)	23 (43.4%)	**<0.001**
Diabetes mellitus	25 (16.7%)	11 (11.3%)	14 (26.4%)	**0.023**

Data presented as median (IQR) or *n* (%). CCS, Charlson comorbidity Score.

**Table 2:** Study group characteristics.

	Study Group (*n* = 53)
Time from the last transplant to hospitalization inyears	5.0 (3.0–8.75)
Type of kidney transplant	
Living donor	7 (13.2%)
Cadaver	46 (86.8%)
Causes of kidney transplantation	
Polycystic kidney disease	7 (13.2%)
Primary glomerulonephritis	4 (7.5%)
Hypertensive nephropathy	3 (5.7%)
Diabetic nephropathy	3 (5.7%)
Secondary glomerulonephritis	2 (3.8%)
Chronic pyelonephritis	1 (1.9%)
Congenital renal hypoplasia	1 (1.9%)
Chronic interstitial nephritis	9 (17.0%)
Juvenile nephropathy	6 (11.3%)
Number of transplantations	
1	47 (90.4%)
2	4 (7.7%)
3	1 (1.9%)

Data presented as median (IQR) or *n* (%).

**Table 3:** Clinical characteristics.

	All Participants (*n* = 150)	Control Group (*n* = 97)	Study Group (*n* = 53)	*p*-Value
Duration of illness before hospitalization, days	7.0 (4.0–10.0)	7.0. (4.0–10.0)	7.0 (4.0–11.0)	0.513
NEWS score				
on arrival	2.0 (1.0–3.0)	2.0 (1.0–3.0)	1.0 (1.0–1.0)	**0.009**
highest *	4.0 (3.0–5.0)	4.0 (3.0–5.0)	3.0 (2.0–5.0)	0.141
COVID-19 disease severity				**<0.001**
mild	25 (16.7%)	11 (11.3%)	14 (26.4%)	**0.023**
moderate	23 (15.3%)	15 (15.5%)	8 (15.1%)	1.000
severe	85 (56.7%)	68 (70.1%)	17 (32.1%)	**<0.001**
critically severe	17 (11.3%)	3 (3.1%)	14 (26.4%)	**<0.001**
Acute kidney injury	19 (12.7%)	1 (1.0%)	18 (34.0%)	**<0.001**
Pneumonia				
on arrival	116 (77.3%)	78 (80.4%)	38 (71.7%)	0.229
during hospitalization *	125 (83.3%)	86 (88.7%)	39 (73.6%)	**0.023**
Hypoxia upon arrival	63 (42.0%)	51 (52.6%)	12 (22.6%)	**<0.001**

Data presented as median (IQR) or *n* (%). NEWS, National Early Warning Score. *—highest during hospitalization period.

**Table 4:** Laboratory data of patients.

	All Participants (*n* = 150)	Control Group (*n* = 97)	Study Group (*n* = 53)	*p*-Value
Leukocyte, ×10^9^/L				
on arrival	5.9 (4.5–8.2)	6.0 (4.5–8.3)	5.6 (4.3–7.7)	0.589
highest *	9.4 (7.3–13.2)	9.4 (7.4–11.5)	11.4 ± 5.9	0.233
Neutrophil, ×10^9^/L				
on arrival	4.2 (3.0–6.3)	4.3 (2.7–6.3)	4.1 (3.3–6.4)	0.827
highest *	6.9 (4.9–10.8)	6.7 (4.5–9.5)	8.1 (5.4–12.7)	**0.018**
Lymphocyte, ×10^9^/L				
on arrival	0.9 (0.7–1.3)	1.0 (0.8–1.5)	0.7 (0.6–1.0)	**<0.001**
lowest **	0.8 (0.6–1.1)	0.9 (0.7–1.3)	0.5 (0.2–0.7)	**<0.001**
CRP, mg/L				
on arrival	53.1 (18.2–106.1)	63.9 (18.3–130.7)	40.4 (17.5–78.7)	0.081
highest *	75.9 (32.1–147.4)	85.5 (31.7–143.6)	70.8 (35.8–200.8)	0.594
IL-6, ng/L				
on arrival	27.7 (12.6–48.3)	27.2 (11.7–52.4)	28.5 (12.6–40.8)	0.545
highest *	31.6 (14.2–54.8)	31.8 (14.4–55.1)	30.3 (12.6–51.5)	0.812
Ferritin, μg/L				
on arrival	421.5 (186.0–917.8)	362.0 (174.5–934.6)	539.0 (192.0–928.0)	0.530
highest *	528.4 (214.8–1050.8)	478.0 (213.2–934.6)	558.0 (219.0–1202.0)	0.376
LDH, U/L				
on arrival	278.0 (213.0–347.0)	285.0 (219.5–367.0)	261.0 (202.3–312.5)	**0.030**
highest *	282.0 (22.3–370.5)	289.0 (223.0–380.0)	267.0 (213.0–345.0)	0.168
D-dimers, μg/L				
on arrival	397.5 (235.0–642.5)	395.0 (235.0–595.0)	405.0 (237.5–800.0)	0.579
highest *	457.5 (253.8–816.3)	400.0 (240.0–630.0)	515.0 (285.0–1067.5)	0.095
Creatinine, μmol/L				
on arrival	92.8 (69.8–141.5)	77.2 (64.0–97.1)	171.5 (121.2–259.2)	**<0.001**
highest *	95.5 (75.0–159.2)	85.0 (66.7–97.3)	180.0 (129.7–280.7)	**<0.001**
eGFR (CKD-EPI), ml/min/1.73 m^2^				
on arrival	67.03 ± 28.5	87.1 (68.1–98.3)	39.4 ±18.4	**<0.001**
lowest **	60.59 ± 27.2	76.0 (60.7–90.0)	31.0 (20.0–48.3)	**<0.001**
Urea, mmol/L				
on arrival	5.8 (4.3–9.7)	5.0 (3.7–6.3)	10.8 (7.2–17.9)	**<0.001**
highest *	7.5 (5.0–12.0)	5.6 (4.4–7.5)	14.9 (9.7–25.2)	**<0.001**
LNR	0.2 (0.1–0.4)	0.3 (0.1–0.5)	0.2 (0.1–0.3)	**0.020**

Data presented as median (IQR), mean (SD), or *n* (%). CRP, C-reactive protein. IL-6, interleukin-6. LDH, lactate dehydrogenase. eGFR, estimated glomerular filtration rate. LNR, absolute lymphocyte to neutrophil ratio. *—highest during hospitalization; **—lowest during hospitalization.

**Table 5:** Treatment and outcomes.

	All Participants (*n* = 150)	Control Group (*n* = 97)	Study Group (*n* = 53)	*p*-Value
Duration of hospitalization, days	12.0 (10.0–17.0)	12.0 (10.0–16.0)	12.0 (9.0–18.0)	0.810
Oxygen therapy	105 (70.0%)	73 (75.3%)	32 (60.4%)	0.065
ICU	15 (10.0%)	3 (3.1%)	12 (22.6%)	**<0.001**
ICU: HighFlow	15 (100.0%)	3 (100.0%)	12 (100.0%)	-
ICU: NIV	7 (46.7%)	0 (0.0%)	7 (58.3%)	0.200
ICU: IMV	8 (53.3%)	1 (33.3%)	7 (58.3%)	0.569
Remdesivir	47 (31.3%)	34 (35.1%)	13 (24.5%)	0.202
Dexametason	106 (70.7%)	65 (67.0%)	41 (77.4%)	0.196
Antibiotic therapy	108 (72.0%)	66 (68.0%)	42 (79.2%)	0.184
Modification of immunosuppressive treatment	-	-	50 (94.3%)	
Outcomes				**<0.001**
Death	11 (7.3%)	1 (1.0%)	10 (18.9%)	
Recovered	139 (92.7%)	96 (99.0%)	43 (81.1%)	

Data presented as median (IQR) or *n* (%). ICU, intensive care unit. NIV, non-invasive ventilation. IMV, invasive mechanical ventilation.

**Table 6:** Risk factors associated with lethal outcome.

	Univariate Regression Analysis HR (95% CI)	*p*-Value	Multivariate Regression Analysis HR (95% CI)	*p*-Value
Age, years	1.01 (0.95–1.06)	0.860		
CSS	1.18 (0.95–1.45)	0.133	1.27 (0.88–1.85)	0.206
Transplantation	11.23 (1.39–90.99)	**0.024**	2.95 (0.26–33.40)	0.383
NEWS value on arrival	1.16 (0.85–1.60)	0.346		
Pneumonia	30.19 (0.04–25,159.76)	0.321		
Hypoxemia	2.49 (0.60–9.60)	0.216		
Creatinine on arrival, μmol/L	1.00 (1.00–1.01)	0.608		
Urea, mmol/L	1.05 (1.00–1.09)	0.060	0.99 (0.91–1.08)	0.822
Lymphocytes on arrival, ×10^9^/L	0.42 (0.10–1.83)	0.251		
LDH on arrival, U/L	1.00 (1.00–1.01)	0.246		
LNR on arrival,	0.20 (0.01–5.79)	0.344		
Oxygen therapy	31.51 (0.04–27,536.70)	0.318		
Treatment in the ICU	32.46 (3.80–277.16)	**<0.001**	20.71 (2.01–213.33)	**0.011**

CCS, Charlson comorbidity score. LDH, lactate dehydrogenase. LNR, absolute lymphocyte to neutrophil ratio. ICU, intensive care unit.

## Data Availability

The data presented in this study are available on request from the corresponding author if a proper procedure would be carried out. The data are not publicly available because it might compromise the privacy of the research participants.
